# Computational Study of Human Head Response to Primary Blast Waves of Five Levels from Three Directions

**DOI:** 10.1371/journal.pone.0113264

**Published:** 2014-11-19

**Authors:** Chenzhi Wang, Jae Bum Pahk, Carey D. Balaban, Mark C. Miller, Adam R. Wood, Jeffrey S. Vipperman

**Affiliations:** 1 Department of Mechanical Engineering and Materials Science, University of Pittsburgh, Pittsburgh, Pennsylvania, United States of America; 2 Department of Chemical & Petroleum Engineering, University of Pittsburgh, Pittsburgh, Pennsylvania, United States of America; 3 University of Pittsburgh, Pittsburgh, Pennsylvania, United States of America; Tel Aviv University, Israel

## Abstract

Human exposure to blast waves without any fragment impacts can still result in primary blast-induced traumatic brain injury (bTBI). To investigate the mechanical response of human brain to primary blast waves and to identify the injury mechanisms of bTBI, a three-dimensional finite element head model consisting of the scalp, skull, cerebrospinal fluid, nasal cavity, and brain was developed from the imaging data set of a human female. The finite element head model was partially validated and was subjected to the blast waves of five blast intensities from the anterior, right lateral, and posterior directions at a stand-off distance of one meter from the detonation center. Simulation results show that the blast wave directly transmits into the head and causes a pressure wave propagating through the brain tissue. Intracranial pressure (ICP) is predicted to have the highest magnitude from a posterior blast wave in comparison with a blast wave from any of the other two directions with same blast intensity. The brain model predicts higher positive pressure at the site proximal to blast wave than that at the distal site. The intracranial pressure wave invariably travels into the posterior fossa and vertebral column, causing high pressures in these regions. The severities of cerebral contusions at different cerebral locations are estimated using an ICP based injury criterion. Von Mises stress prevails in the cortex with a much higher magnitude than in the internal parenchyma. According to an axonal injury criterion based on von Mises stress, axonal injury is not predicted to be a cause of primary brain injury from blasts.

## Introduction

The intensive usage of improvised explosive devices (IED) as weapons in the ongoing conflicts and terrorist activities around the world has led to the prevalence of blast-induced traumatic brain injury (bTBI). Unlike previous wars, improved battlefield medical treatment and personnel armor reduced the mortality rate of the service members who have been wounded by blasts [5],[22]. However, the increased survival rate of blast victims results in the increased number of people suffering from bTBI. According to the report of Warden et al. [41] in WRAMC, 56% of the blast-exposed patients admitted to Walter Reed Army Medical Center (WRAMC) were diagnosed with moderate to severe TBI, while the rest were considered to have mild TBI. A RAND Corporation report estimates that about 19.5% of the deployed US Armed forces in Iraq and Afghanistan potentially received a TBI [35]. Explosive detonations generate an expanding blast wave characterized by an initial impulse of atmospheric overpressure followed by an exponential decay to an under-pressure which causes a reverse blast wind toward the under-pressure area. The primary blast injury is due to the direct effects of the blast wave force on the human head; the secondary blast injury is induced by the impact of the blast-propelled segments; the tertiary blast injury results from the collision of blast-propelled people with the ground or a rigid wall; the quaternary blast injury results from the toxic gases or heat generated from blast, and includes all the other injury effects [22].

It is believed that primary blast waves traversing across a human head can cause tissue damage throughout the brain, therefore inducing mild, moderate, and severe brain injury [7],[19],[22],[34]. Headache, hearing impairment and balance dysfunction are the major symptoms of mild TBI that occur acutely after exposure to low level blasts. Mild TBI impedes its sufferers return to normal activities, and can persist chronically [14]–[16],[17],[37]. Blast over-pressurization waves instantaneously increase the pressure in body tissues, forming stresses in the intracranial tissues. Once the resultant stresses in the intracranial tissues exceed the tolerable threshold, traumatic brain injury occurs. Cerebral contusion, subdural hematoma (SDH), and diffuse axonal injury (DAI) are the major probable injury types of bTBI [19],[34]. Cerebral contusion and diffuse cerebral edema have been reported in some clinical cases of blast victims [1],[3]. Clinical case reports have also shown that intracranial hemorrhage including subdural hemorrhage and subarachnoid hemorrhage happened following IED blasts or industrial explosion accidents [22],[23],[26]. Mac Donald et al. [23] validated the hypothesis that bTBI can involve axonal injury by using diffusion tensor imaging (DTI) to detect traumatic axonal injury among 63 military personnel who had a clinical diagnosis of mild, uncomplicated blast-related TBI. However, this study did not determine the contribution of primary blast exposure as compared with that of other types of injury because none of the subjects with TBI had isolated primary blast injury. In sum, the difficulty of gathering the subjects of isolated primary bTBI hinders research to determine the mechanisms of primary bTBI from clinical studies.

Several researchers have used animal studies to elucidate the pathophysiological characteristics of bTBI. In the study by Saljo et al. [31], anesthetized pigs were subjected to controlled blasts in order to simulate real battlefield blast scenarios including the explosions generated by howitzer, bazooka, automatic rifle in the free field, the explosions in an enclosure, and underwater blasts. Pressure-time histories recorded by the transducers in the porcine brains for the howitzer experiments were found to have a strong similarity with those in air. Macroscopic examinations revealed that subdural hemorrhages occurred in 21% of the animals exposed to the automatic rifle in free field and in 7% of those exposed to the bazooka. Histological examination of porcine brains also revealed that small parenchymal and subarachnoid hemorrhages predominated in the occipital lobe, cerebellum, and medulla oblongta/lower brainstem. Bauman et al. [2] conducted blast experiments on pigs in a number of environments, including a bi-directionally open-ended blast tube, a surrogate of a HUMVEE crew-compartment, and a building that consisted of four walls without a roof. The blast tube included the heavy-walled driver chamber to immobilize the chemical explosives, the expansion cone, and the test section in which pig was restrained to sustain blasts from different distances. Edema, intracranial hemorrhage, and vasospasm were revealed to be the most salient pathophysiological characteristic of bTBI. Rafaels et al. [29] exposed the heads of twelve male New Zealand White rabbits, whose bodies were protected by test fixtures, to shock waves generated by a helium-driven shock tube. Histological examination revealed subdural and subarachnoid hemorrhages in the nonresponsive respiratory-arrested specimens. Cerebral contusion, subdural hemorrhage, and subarachnoid hemorrhage occurred together in all non-surviving specimens.

Neither clinical nor basic animal research studies have fully explained the injury mechanisms of primary bTBI. Moreover, the results of animal studies do not translate directly to the injury mechanisms of human bTBI due to the geometrical scaling and anatomical structural differences between human and either rodents or larger mammals. The challenge in determining the mechanisms of primary bTBI comes from the difficulties in measuring the injury process of bTBI *in vivo* and from the difficulty of distinguishing the primary brain injury from other types of injuries in the chaotic environment of the battlefield, industrial accidents or terrorist incidents. The development of protection devices and medical treatment requires a clear understanding of the injury mechanisms including the brain responses to blast waves, brain sensitivities to various blast factors, and the injury thresholds to specific injury types.

In biomechanics, the finite element (FE) modeling and simulation approach is able to predict responses of complex biological structures under various types of mechanical loadings, therefore helping to understand biomechanical details and to increase research efficiency. There have been a number of computational attempts to investigate the mechanics of human head under blast loadings by using finite element head models developed by CT and MRI medical images [4],[25],[36]. Taylor and Ford [36] developed a FE head model consisting of skull, white matter, gray matter, CSF and air in sinuses and simulated the direct head exposure to a blast wave of 1.3 MPa peak pressure from anterior, posterior, and lateral directions for 2 ms. Elevated pressure, volumetric tension, and deviatoric stress in focal areas of the brain were revealed. Chafi et al. [4] simulated the blast-head interactions of three different TNT amounts for up to 5 ms using a FE head model comprised of brain, falx and tentorium, CSF, dura matter, pia matter, skull bone, and scalp. They predicted that significant positive and negative pressures were alternating at the coup and contrecoup sites. Moore et al. [25] developed an FE head model for three simulations of a 5.2 atmosphere pressure blast, an 18.6 atmospheric pressure blast, and a 5 m/s impact. The highest stresses were located at the right temporal region where the blast wave was incident. Grujicic et al. [12] developed a finite element head model assembled with a helmet to study the mitigation effect of the advanced combat helmet against blast. Ganpule et al. [10] simulated the blast wave-head interactions using a developed FE head model consisting of the skin, skull, subarachnoidal space, and brain. They also built a shock tube to test the surface effects of blast waves impinging upon a dummy head.

Despite these previous investigations of the mechanics of blast wave-brain interaction by computational modeling, there has been less emphasis on the responses of the brain and other cranial contents (e.g., veins) to blast waves of different loading factors such as blast wave intensities and exposure directions. The purpose of this study is to understand the variation of the pressure and shear stress responses of human brain due to three exposure orientations and five blast levels. This variation influences the resulting injury pattern and severity. An anatomically correct FE human head model is constructed from MRI and is validated in order to perform the simulations of blast wave-head interactions, which are modeled by a coupled Euler-Lagrange method in a commercial explicit finite element code. The head is exposed to the blast waves coming from anterior, right lateral, posterior orientations at one meter distance to form three scenarios of blast-head interaction simulations under free-field blast conditions. Each scenario has the identical five blast wave levels corresponding to the five impinging blast overpressures (BOPs) at the blast incident side of the head. The various aspects of the mechanics of blast wave/head interactions including the sensitivities of brain responses to different blast loading factors, the transmission pathways of intracranial pressure waves, and the location variations of response factors are investigated. The intracranial pressures and von-Mises stresses at several cerebral locations are predicted and serve as the main brain response factors to help understand the mechanism, injury occurrence, and potential injury severity of bTBI. The understanding from this study is essential for improving the medical treatment and developing the next generation of protective equipment that will better protect people against bTBI.

## Methods

### Finite element head modeling

**Figure 1:**
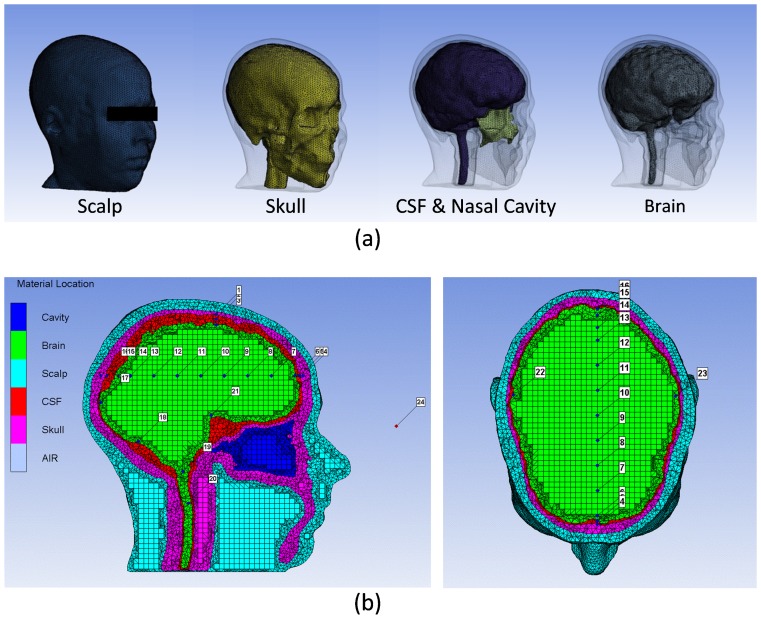
Finite element head model showing (a) progressively more internal anatomical layers from left to right (b) Locations of gauges.

The three-dimensional head model is developed from magnetic resonance images (MRIs) obtained from the International Consortium for Brain Mapping database [18]. Each image layer is segmented into its components of scalp, skull, brain matter, CSF, and nasal cavity using BrainSuite 11a software [32] and MIMICS 13.0 software (Mimics 13.0, Materialise, Leuven, Belgium). This three-dimensional head model is based on the same MR images used by the previous studies [38]–[39], while it is improved by segmenting the scalp, the nasal cavity, and the skull layer between the nasal cavity and the CSF. The head geometric model is discretized to include linear tetrahedral elements, linear hexahedral elements, linear pyramid elements by using ANSYS ICEM CFD (ANSYS, Inc., Canonsburg, PA), which provides powerful meshing algorithms to discretize highly complex geometry into a high-quality grid. The fully developed finite element head model includes the 3D finite element models of scalp, skull, cerebral spinal fluid (CSF), nasal cavity, and brain (Figure 1a). The Dura mater, a semi-rigid layer firmly attached to the skull, is modeled together with the skull since it is too thin to be distinguished from the skull. The whole FE human head model consists of 607,310 elements including the whole scalp FE model (280,175 elements), the whole skull FE model (154,658 elements), the CSF FE model (54,878 elements), the nasal cavity FE model (12,024 elements), and the whole brain FE model (105,575 elements). In order to monitor the mechanical responses of the head to blast waves, 23 virtual gauge points are placed inside the head (Figure 1b). Particularly, on the superior sagittal sinus (SSS) region, gauges No. 1, No. 2 and No. 3 are placed in the skull, CSF, and cortex respectively, in order to record the complete mechanical responses of the three distinct tissue layers surrounding the SSS. Gauges No. 4 to No. 16 are placed in the head at various locations along the anterior-posterior direction in the mid-sagittal plane, in order to track the evolvement of the mechanical responses along the pathway of the blast wave. From gauge No. 6 to gauge No. 13, a distance of 20 mm is specified. Gauge No. 24 is fixed near the head model in the air to track the pressure-time histories of the blast waves impinging on the head without location change even if the exposure orientation to blast switches.

### Material modeling

Five constituents of the head, the ambient air, and the TNT material are represented by the constitutive relations and the associated material constants adopted from literature. The constitutive relation of scalp and nasal cavity is linear elastic and is described by bulk modulus and shear modulus, representing volumetric and deviatoric responses respectively. The material constants of the scalp and the nasal cavity are chosen from the bTBI study of Moore et al. [25]. Bulk modulus *K*, density ρ, and shear modulus *G* of the scalp are 34.7 MPa, 1.04 g/cm^3^, and 5.88 MPa respectively. The material property of the scalp model is also identical to that of scalp/skin in other literatures [4],[10],[42]. Bulk modulus, density, and shear modulus of the nasal cavity are 2.19 GPa, 1.04 g/cm^3^, and 225.3 Pa, respectively.

Skull is composed of bone material, and it has the highest rigidity among all the anatomical parts of the head. In order to capture the large volumetric compressions of the skull that may arise in the severe blast loading events, the dilatational part of the skull constitutive model is modeled by the Mie-Gruneisen equation of state (EOS) expressed by:
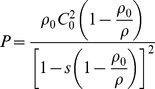


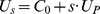
 where *P* is pressure, ρ is the density, *U_s_* is the shock velocity, *U_P_* is the particle velocity. *ρ_0_*, *C_0_* and *s* are the material constants. For skull, the reference density is *ρ_0_* = 1.412 g/cm^3^, the parameter *C_0_* is 1,850 m/s, and the dimensionless parameter *s* is 0.94 [25]. Similar to the material modeling of the scalp and nasal cavity, the deviatoric response of the skull is only described by a single material parameter, shear modulus *G*, which is calculated to be 2.664 GPa by the corresponding Young's modulus *E* and Poisson's ratio *ν* from literature [25].

CSF is known as a Newtonian fluid that fills the subarachnoid space and ventricular system. The global CSF model is assigned the properties of water since the density and viscosity of CSF are very similar to that of water [42]. The hydrostatic property of CSF is characterized by the Mie-Gruneisen equation. *C_0_* is 1,647 m/s *ρ_0_* is 0.998 g/cm3, and the parameter *s* = 1.921 [6]. Since the CSF model is modeled by tetrahedral elements, which are only supported in Lagrangian formulations in AUTODYN 14.0, a very low shear modulus of 500 Pa, adopted from the study of Zhang et al. [42], is assigned to CSF to associate the hydrostatic property.

The cerebrum, cerebellum, brainstem and spinal cord are three major structural divisions of the whole brain. They are formed by neurons, glia cells and cells associated with vasculature and meninges. White matter consists of primarily of bundles myelinated neuronal axons and associated glia; gray matter consists predominantly of neuronal cell bodies and dendrites (with associated glia), interspersed with axons that may or may not be myelinated. The material properties of white and gray matters differ, but both exhibit complex mechanical responses such as hyperelasticity and viscoelasticity. White matter properties include regional differences of material behavior due to the variability of the orientation of nerve fiber bundle directions. Gray matter can be viewed as isotropic. In this study, the material modeling of white matter and gray matter are the same due to the integrated geometrical modeling of the two types of matter. Since white matter accounts for a higher portion of brain than gray matter does, the constitutive law and material constants of the whole brain model are assigned as those of the white matter reported by Zhang et al. [42], who determined the associate material constants based on *in vitro* vibration tests of human brain tissue [33]. The bulk modulus *K* (2.19 GPa) models the dilatational response of the whole brain model, and the deviatoric response is modeled by a linear viscoelastic constitutive law expressed by the time-dependent shear modulus:
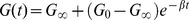
where *G_0_* is the short-term shear modulus (41 KPa), *G_∞_* is the long-term shear modulus (7.8 KPa), β is the viscous decay constant (700 *s^−1^*), and *t* is time. The constitutive laws and the associated material constants used for the finite element head model are summarized in Table 1.

**Table 1:** Constitutive Laws and Associated Material Constants of FE Head Model.

Component	Constitutive law	Material Constants
*Scalp*	*Linear Elastic*	*K = 34.7 MPa, G = 5.88 MPa, ρ = 1.04 g/cm^3^*
Nasal cavity	Linear Elastic	*K* = 2.19 GPa, *G* = 225.3 Pa, *ρ* = 1.04 g/cm^3^
Skull	Mie-Gruneisen EOS	*C_0_* = 1,850 m/s, *s* = 0.94, *ρ_0_* = 1.412 g/cm^3^, *G* = 2.664 GPa
CSF	Mie-Gruneisen EOS	*C_0_* = 1,647 m/s, *s* = 1.921, *ρ_0_* = 0.998 g/cm^3^, *G* = 500 Pa
Brain	Viscoelastic	*K* = 2.19 GPa, *ρ* = 1.04 g/cm^3^, *G_0_* = 41 KPa, *G_∞_* = 7.8 KPa, β = 700 s^−1^

The constitutive modeling of TNT follows the Jones-Wilkins-Lee (JWL) EOS [21], and the constants are adopted from Lee et al. [20]. The constitutive model of the air domain is characterized by the ideal gas EOS, which has material constants picked from Rogers and Mayhew [30]. ANSYS Autodyn provides the standard material library of TNT and ideal gas for direct application.

### Simulation methods

The FE head model is imported into the engineering computational analysis software ANSYS Autodyn (ANSYS, Inc., Canonsburg, PA) for the validation simulation and the blast-head interaction simulations. ANSYS Autodyn provides finite volume solvers for fast transient computational fluid dynamics (CFD) to simulate blast wave propagation in gas or fluid, explicit finite element solvers for analysis of solid structure, and the fluid-structure interaction (FSI) algorithms for the coupling between the blast wave and solid structure. The anatomically correct human FE head model is validated using the experimental data of a published experiment for frontal impact on a cadaveric head [27]. In order to reproduce Nahum's experiment, the measured input force of this test is applied to the center area (2,526 mm^2^) of the forehead of head model in the anterior-posterior direction with 45^o^ inclined to the horizontal as a form of a distributed load. The peak load is 7,000 N and has an 8 ms duration. The pressure-time histories are recorded at the same frontal cortex and the posterior fossa areas as in the experiment during the validation simulation. The frontal peak pressure of the validation simulation is almost the same as the experiment, while the peak negative pressure at posterior fossa of the simulation is 7.74% less than that of the experiment. So, the present FE head model is considered accurate to be used for finite element simulations. Since the FE head model is not validated against any cadaveric head experiments under blast loadings, it currently can only be claimed as partially validated.

**Figure 2:**
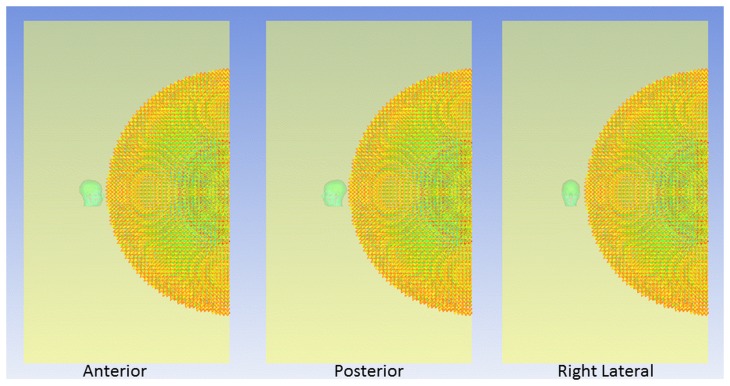
Three scenarios of blast wave-head interactions according to the three exposure orientations of the FE head model to horizontal blast wave: anterior, posterior, and right lateral.

For the simulations of blast wave-head interactions, a 3 m by 1.8 m by 3 m cuboid air space is established to model the air domain through which blast wave propagates. The simulation of the blast wave initiation and propagation in the air domain is processed by the Eulerian solver. Eulerian hexahedron elements of edge length 30 mm are used to mesh the air space. The Eulerian grid maintains its shape while the fluid moves within the grid, thus it is suitable for the numerical simulation of blast wave propagation. The head model is formulated with Lagrangian elements, which can deform with the material. Solid material undergoes relatively smaller deformations than the fluid media, so Lagrangian elements can better simulate the mechanical responses of a human head. Simulations of human head exposure to blast waves are performed from three principal orientations of the explosion, i.e. anterior, posterior, and right lateral orientations. Every exposure orientation has five blast-head simulations corresponding to five different blast intensities produced by the simulated detonations of 250 g, 300 g, 350 g, 400 g, and 450 g center-ignited TNT spherical explosives at one meter distance from the head. The head model is immersed in the cuboid air domain at the location where its surface is 1 meter away from the explosive center, which is located at the center of the right lateral surface of the air domain for every exposure direction (Figure 2). Before the onset of the blast simulations, the internal energy density of the air domain is set to ambient conditions such that the atmospheric pressure can return to a standard atmosphere after the blast energy fully dissipates from the air space. In order to save the computational expense of blast simulation, ANSYS Autodyn provides a tool called "remapping" that allows us to first simulate a one-dimensional blast model in which blast wave can propagate a certain time, and then to remap the result of the one-dimensional model into the three-dimensional air domain to construct a complete three-dimensional spherical blast wave. The radius of every TNT explosive is calculated from the mass in order to set up the corresponding one-dimensional TNT air blast model, in which the length of the one-dimensional air is set to one meter i.e. the distance between the blast center and the human head. An open boundary condition, enabling high pressure waves to propagate out of the space without reflection, is assigned to the surfaces of the air space except the right surface where the detonation center is located. The duration of all the fifteen blast wave-head simulations is set to 5 ms, which is sufficient for the blast waves to completely traverse the head and to dissipate from the air space thoroughly. The simulations are designed to model the scenarios that a soldier experiences in an open-field environment, including blast waves of five distinct blast intensities that are applied from three principal directions. The FSI between the blast waves and the head model are modeled by a fully coupled Euler-Lagrange interaction algorithm embedded in ANSYS Autodyn.

## Results

The predicted intracranial pressure and cerebral von-Mises stress are chosen as the response factors of the brain for a given blast wave loading. These factors have distribution contours and time histories at locations of interest in the head that vary with the blast overpressure (BOP) levels and orientations of the blast wave relative to the head. The distribution patterns of the intracranial pressure and the von-Mises stress of the brain model can then be used to estimate the likelihood and severity of injury in different brain regions. Thus, the influences of the BOPs on the blast incident side and the exposure orientations on the injury severities of cerebral contusion at various cerebral regions of interest are assessed based on the corresponding levels of the intracranial pressures. The extent of diffuse axonal injury (DAI) is also estimated from the focal von-Mises stresses.

### Brian pressure response to blast wave

**Figure 3:**
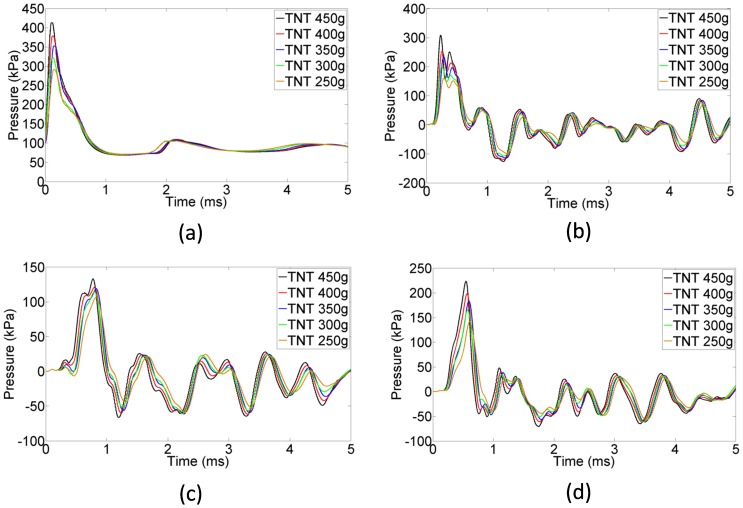
Pressure time histories of the anterior blast-head simulations: (a) impinging blast waves (b) frontal cortex (c) posterior margin of the tentorium cerebelli (d) lower brainstem.

The pressure waveform of a blast wave in open-space air is a Friedlander wave [9], which begins with a sharply rising over-pressure followed by an under-pressure causing a brief vacuum, and finally returning to the normal atmospheric pressure after subsidence of the blast energy. The shock front of the three-dimensional spherical blast wave expands and propagates purely along the radial direction. The magnitudes of the propagating BOPs are several times that of the standard atmospheric pressure. As the TNT weight increases, the peak BOP at the incident side increases as well. The 250 g, 300 g, 350 g, 400 g, and 450 g TNT charges have impinging BOPs of 291 kPa, 321 kPa, 349 kPa, 379 kPa, and 413 kPa, respectively for every head orientation (Figure 3a). The pressure time histories of the various intracranial locations exhibit a pattern similar to that of the ambient pressure-time history: the intracranial pressures begin with sharply rising and falling to form a pulse, which thereafter returns to zero pressure. Figure 3 (panels b, c, and d) shows the pressure-time histories of the frontal cortex, posterior margin of the tentorium cerebelli, and lower brainstem sites for the anterior blast scenario. Higher peak BOPs caused higher peak positive pressures within the brain for most intracranial locations. The intracranial pressure wave attenuates during its propagation from the proximal to distal side of the brain. Taking the 300g TNT case of the anterior exposure as an example, the peak positive pressures at the frontal cortex and occipital cortex are 201 kPa and 138 kPa, respectively, so the corresponding percentage of attenuation in magnitude is 31%.

The highest and the lowest peak positive pressures exist, respectively, on the proximal and distal cerebral locations to blast waves for all the cases irrespective of exposure orientation. For the anterior exposure scenario, the highest values of peak positive pressures occur at the frontal cortex (gauge No. 6) with the range from 161 kPa to 308 kPa, while the lowest values occur at the midsagittal posterior parietal/superior sagittal sinus area (gauge No. 12) with the range from 87 kPa to 94 kPa. The peak positive pressures of the occipital cortex (gauge No. 14) in the anterior scenario range from 132 kPa to 142 kPa. The peak positive pressures from the gauges No. 6 to No. 12 exhibit an apparent decreasing gradient. The mitigation of the intracranial pressure indicates that the pressure wave traveling in the head has some blast energy being absorbed by the viscoelastic brain tissue. However, the peak positive pressures from the gauges No. 12 to No. 14 exhibit a slight increasing gradient, as the areas of the corresponding coronal cross sections of the brain decrease. The skull chamber is broader in the middle while narrower in the front and back, so the levels of the intracranial pressure waves are reinforced as they travel from the broader cerebral cross-section to the narrower one, as long as the attenuation effects of the brain tissue are secondary to the concentration effect of the narrow region. Other than the peak pressure, using the peak-to-peak pressure as a factor should not be ignored because it may act as a more accurate parameter to predict brain injury than peak pressure. Peak-to-peak pressure varies at different intracranial locations. The highest intracranial peak-to-peak pressure (rapid fluctuation from positive to negative pressure) occurs in the frontal cortex at 0.6s (Figure 3b) and is 432.1 kPa. The highest peak-to-peak pressure in the brainstem (see figure 3d at 0.8s) is 273.5 kPa, which is not much more than the peak pressure. In the tentorium ceribelli, the highest peak-to-peak pressure occurs around 1s (Figure 3c) and is 216.6 kPa.

**Figure 4:**
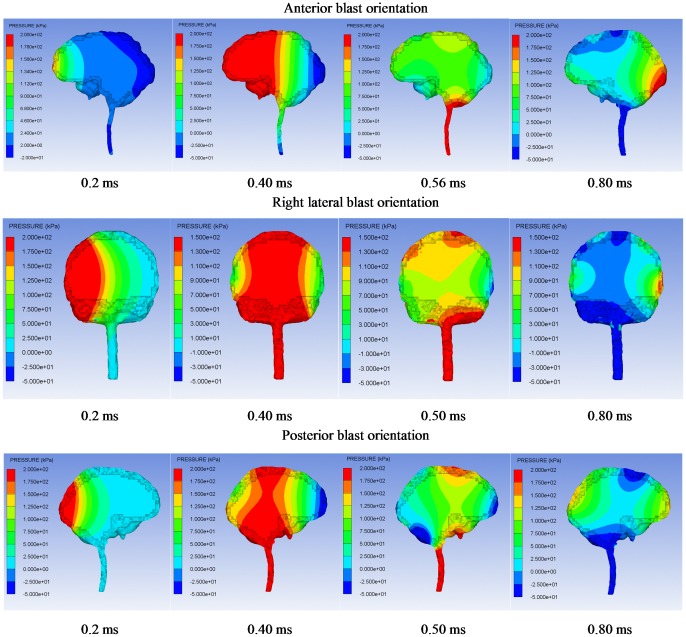
Pressure distributions of the brain in the 400g TNT blast wave-head simulations of all blast orientations.

Figure 4 shows the transmission of the intracranial pressure wave in the brain from 0.2 ms to 0.8 ms for all the blast orientations. For every scenario, the high pressure region of the brain first occurs at the side that is proximal to the blast wave source, then moves to the central cerebral region, and finally arrives at the side which is distal to the blast wave source. The highest pressure always occurs in the cerebral region which is proximal to the blast wave source for every orientation, so the severity of parenchymal injury is expected to be a function of proximity to the blast wave source. Therefore, it is concluded that the propagation of the intracranial pressure wave induced by the direct blast wave transmission into the head is the brain pressure pattern of the bTBI, and this pattern is distinct from that of the impact-induced TBI, i.e. the so-called “coup and contrecoup” pressure phenomenon, in which the high level positive pressure prevails at the impact side while high level negative pressure with similar magnitude prevails opposite the impact site simultaneously.

In the cortex, the right lateral blast scenario has the highest peak positive pressures at the proximal side, i.e. right temporal cortex (gauge No. 22), ranging from 240 kPa to 421 kPa, as compared to 221 kPa to 402 kPa at the occipital cortex (gauge No. 14) of the posterior blast scenario, and to 161 kPa to 308 kPa at the frontal cortex (gauge No. 6) of the anterior blast scenario. The most injurious loading direction for the cortex predicted in this study is very similar to the bTBI simulation study of Taylor and Ford [36], who revealed that the lateral blast was more injurious than the frontal and posterior blasts.

In the brainstem, the posterior blast scenario has the highest peak positive pressures, with a range from 247 kPa to 446 kPa. In the posterior scenario, the pressure levels on the lower brainstem are even higher than that on the occipital cortex, so the lower brainstem in the posterior scenario has the highest intracranial pressures among all the cerebral locations. The intracranial pressure waves propagating into the lower brainstem are reinforced by the concentrating effect of the foramen magnum, which has a narrow region, even though the lower brainstem and the occipital cortex are both proximal to the blast waves. A high-level positive and negative pressure exists at the lower brainstem and spinal cord for every scenario. The intense compressive and tensile pressures as well as their sharp transition in the posterior fossa and foramen magnum, especially for the inferior aspect of the cerebellum, pons, medulla and cervical spinal cord, could make the parenchyma and vasculature particularly vulnerable to injury.

### Brain shear stress response

Von Mises stress is taken as an indicator of the shear stress analysis for the present study. The cortex, brainstem, and spinal cord are found to be the primary locations of high-level shear stresses, and the internal brain parenchyma has the lowest shear stresses (less than 0.1 kPa). It is believed that the density difference between the brain tissue and the CSF stretches the neurons that traverse the interfaces between the areas of different densities. This sliding force between the brain and the CSF causes high-level shear stress at the cerebral surface adjacent to the CSF, whereby the intracranial tissues absorb the kinematic energy of the blast wave transferred to them. The cortico-medullary junction (gray matter-white matter transition) experiences high-level shear stress; hence, axons may be particularly vulnerable to shear stress at this site. The localized shear can cause DAI lesions by stretching, separating, and disrupting the nerve fiber tracts [24],[34].

Like the pressure responses, the pattern of the time-history of von Mises stress remains the same at each intracranial location as the blast strength varies; however, the peak von Mises stress increases with increasing blast strength. For every simulation case, the peak shear stresses in the brainstem and spinal cord are higher than that in the cortex. In the anterior scenario, the highest von Mises stresses of cortex occur at the frontal region (gauge No. 6) with the peak values ranging from 0.73 kPa to 1.10 kPa; for the right-lateral scenario, the highest von Mises stresses in the cortex occur at the right temporal region (gauge No. 22) with the peak values ranging from 1.09 kPa to 1.58 kPa; in the posterior scenario, the highest von Mises stresses in the cortex occur in the right temporal region (gauges No. 22) with the peak values ranging from 0.74 kPa to 1.18 kPa. The anterior scenario has the highest von Mises stresses at the lower brainstem/medulla oblongata with peak values ranging from 1.55 kPa to 2.81 kPa, whereas the right lateral and posterior scenarios have the highest von-Mises stresses in the spinal cord, with the peak values ranging from 1.33 kPa to 2.06 kPa, and from 1.48 kPa to 2.10 kPa, respectively. The spinal cord is not the point of interest of this study, so the injury analysis of DAI does not include the spinal cord.

**Figure 5:**
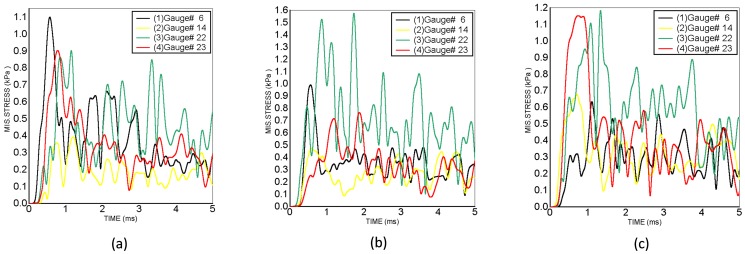
von-Mises stress time histories of the gauge points No. 6 (frontal cortex), No. 14 (occipital cortex), No. 22 (right temporal cortex), and No. 23 (left temporal cortex) for the 450 g TNT blast-head simulation cases: (a) Anterior blast orientation, (b) Right lateral blast orientation, (c) Posterior blast orientation.

As a typical example, the time histories of von Mises stress for the gauges No. 6, No. 14, No. 22, and No. 23 at the cortex in the 450g TNT cases are shown in Figure 5. The von Mises stress-time histories at some of the cortex locations resemble an "impulse-like" pattern, i.e. a similar pattern of the BOP and the intracranial pressure time histories, such as gauges No. 6 and No. 23 in the anterior scenario. The time histories of von-Mises stresses of several cerebral locations continue to develop after 1 ms throughout the whole simulation time, some of them showing a time history pattern quite different from the “impulse-like” pattern. Therefore the pattern of brain von-Mises stress is not unique.

The von-mises stresses of brain show a higher dependency on skull thickness than intracranial pressures. The posterior part of the skull is much thicker than the temporal parts. Although the occipital region is closer to the blast than the temporal regions, in the posterior orientation blast (Figure 5c), the recorded von-mises stresses are highest in the temporal regions instead of the occipital region.

**Figure 6:**
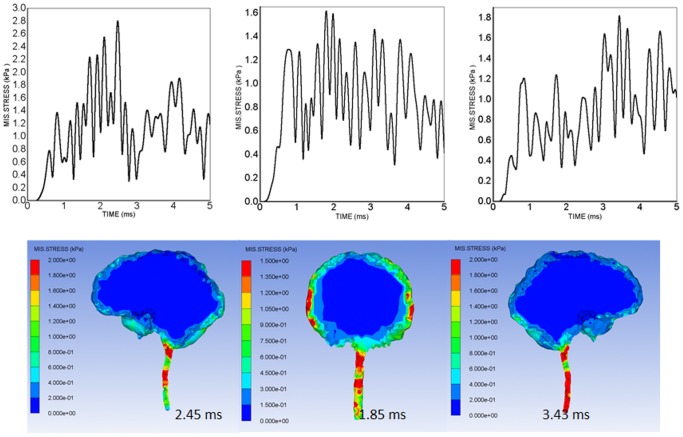
von-Mises stress time-histories of gauge No. 19 at lower brainstem and the associated von-Mises stress distributions showing peak von-Mises stresses in the 450 g TNT blast-head simulation cases: left - anterior orientation (2.45 ms), middle - right lateral orientation (1.85 ms), right - posterior orientation (3.43 ms).

Figure 6 shows the time histories of von Mises stresses on the lower brainstem of the three 450 g TNT cases, along with the corresponding intracranial von Mises stress distributions at the point where the peak von Mises stresses occurred at the lower brainstem. Because the foramen magnum contains the anatomic structures with different densities in this limited space, which could concentrate more energy from the blast, the von Mises stress time histories of the lower brainstem still develop and remain at high levels through the whole simulation time. The concentration of high von Mises stresses on the cerebral tissues adjacent to the CSF is also clearly revealed in every blast orientation.

## Discussion

The intracranial pressure pattern at the blast proximal site and distal site predicted by the present study is quite different from the "coup and contrecoup" intracranial pressure pattern observed for a human head sustaining impact or acceleration loadings. It is concluded that the direct blast wave propagation into the brain (and not the blast-induced impact) generates the propagating intracranial pressure wave. This conclusion is similar to that described by others in the literature [25],[28],[36], which excluded the “coup and contrecoup” pattern from the intracranial pressure patterns of bTBI too. But the distribution pattern of the blast-induced intracranial pressure is still controversial. Chafi et al. [4] predicted that the alternating compression and tension occurred at both of the coup and contrecoup sites due to the brain translational and rotational movement which might be attributed to the blast-induced impact. Ganpule et al. [10] predicted that the typical “coup and contrecoup” pattern occurred at the blast proximal site and the distal site, and that highest positive pressure occurred at the inner parenchyma instead of the proximal cerebral site. In the present study, on both the proximal side and distal side of the brain exposed to blasts, the positive pressures are much more significant than the negative pressures. The main peak positive pressure and the subsequent peak negative pressure occurred over a very short period of time (less than 0.8 ms). This sudden transition from high-level compression to tension might introduce an additional mechanism of axonal injury. This hypothesis also agrees with that which was proposed by Taylor and Ford [36].

**Figure 7:**
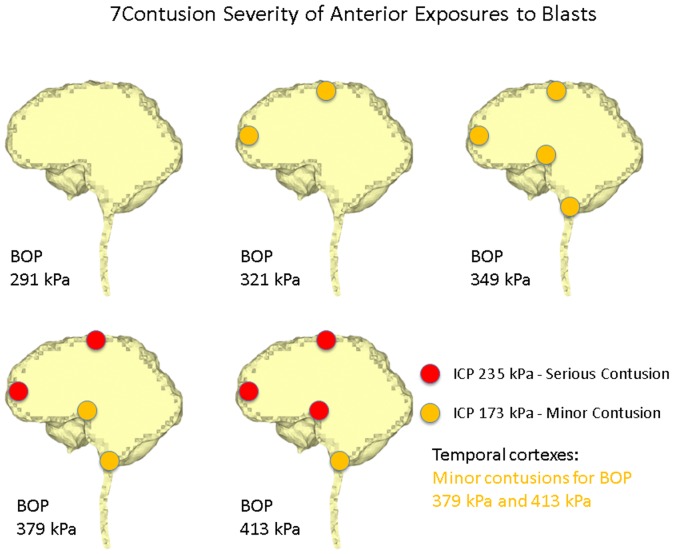
Predicted contusion injury severities at typical cerebral sites with respect to impinging BOPs for the anterior blast scenario.

**Figure 8:**
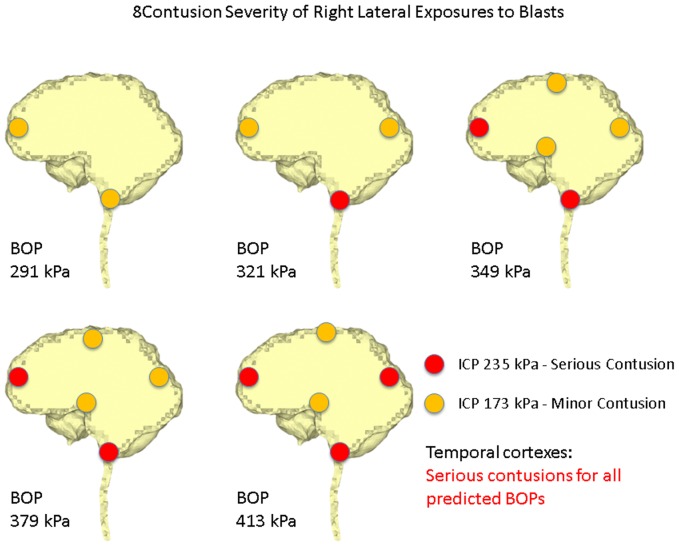
Predicted contusion injury severities at typical cerebral sites with respect to impinging BOPs for the right lateral blast scenario.

**Figure 9:**
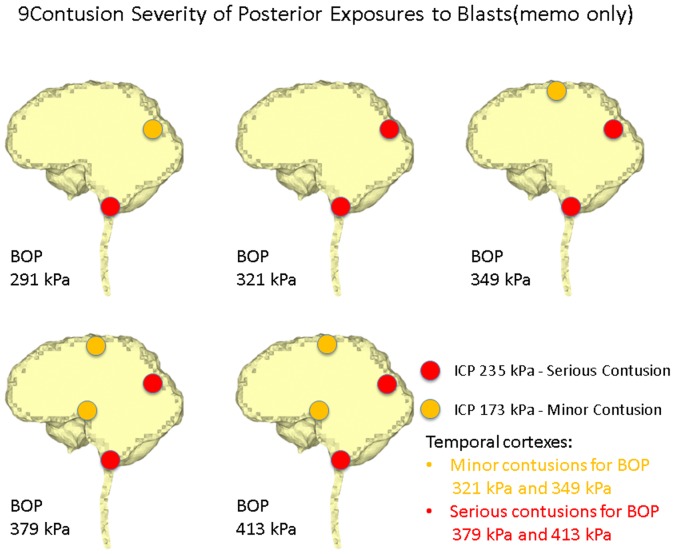
Predicted contusion injury severities at typical cerebral sites with respect to impinging BOPs for the posterior blast scenario.

**Figure 10:**
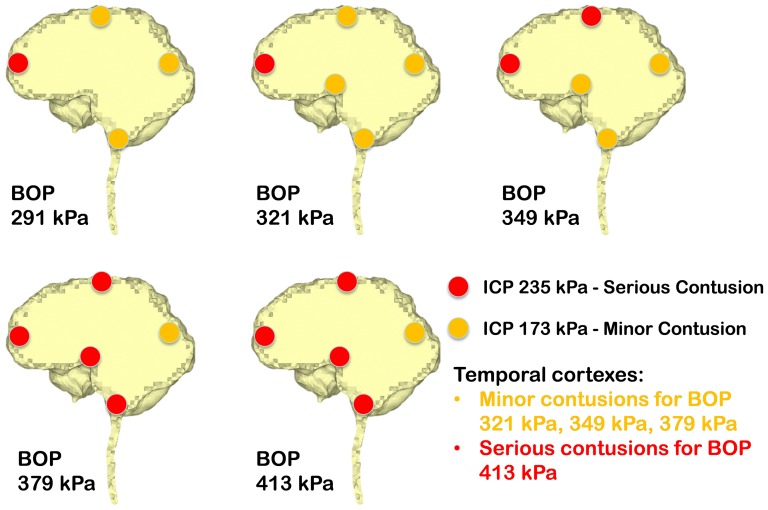
Predicted contusion injury severities from difference of peak positive pressure and peak negative pressure at typical cerebral sites with respect to impinging BOPs for the anterior blast scenario.

**Figure 11:**
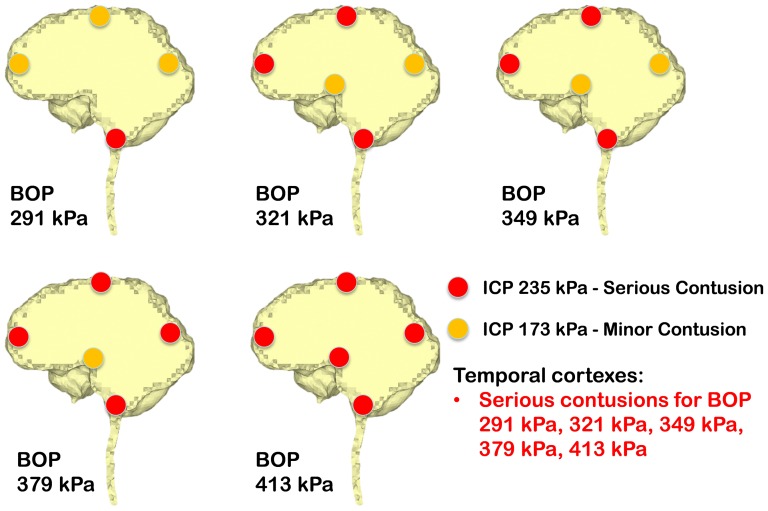
Predicted contusion injury severities from difference of peak positive pressure and peak negative pressure at typical cerebral sites with respect to impinging BOPs for the right lateral blast scenario.

**Figure 12:**
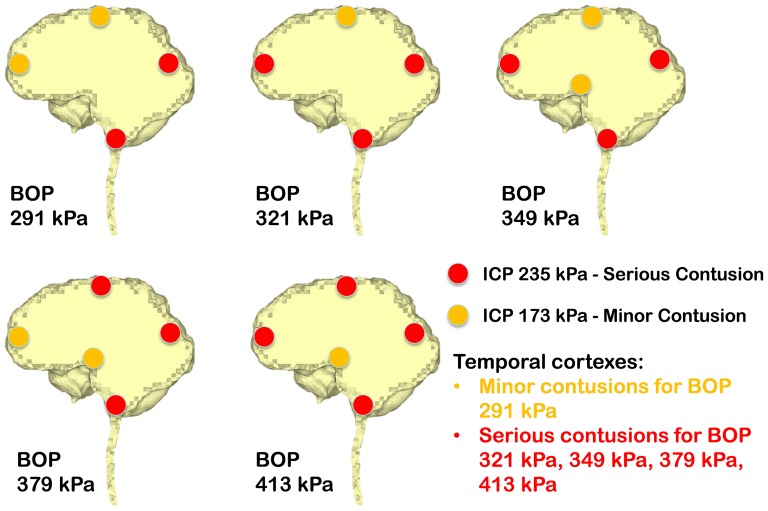
Predicted contusion injury severities from difference of peak positive pressure and peak negative pressure at typical cerebral sites with respect to impinging BOPs for the posterior blast scenario.

Ward et al. [40] proposed an intracranial pressure injury index to evaluate the occurrence and the injury severity of cerebral contusion. According to this intracranial pressure tolerance criterion/index, the peak intracranial pressure of more than 235 kPa would induce serious cerebral contusion, while minor or no brain injury would occur when the intracranial pressure was below 173 kPa.etween 173 kPa and 235 kPa, minor contusion or cortex hemorrhage would occur. Predicted intracranial pressure responses at the proximal side (frontal cortex) for all the blast simulation cases of the anterior exposure, except the 250 g TNT case, are higher than 173 kPa, and the 400 g TNT case has 252 kPa peak pressure which is more than the 235 kPa serious injury threshold. Therefore, people experiencing the anterior blast waves with the impinging BOP at the blast incident side from 321 kPa to 349 kPa would risk suffering a minor contusion or cerebral cortex hemorrhage, while people experiencing the anterior blast wave with the impinging BOP at the blast incident side with more than 379 kPa are expected to have serious cerebral contusion. All cases of right lateral blast exposures have peak positive pressures of more than 235 kPa at the cortex region proximal to the blast (right temporal cortex), indicating that subjects experiencing a lateral blast wave with the impinging BOP of more than 291 kPa at the blast incident side would have serious cerebral contusion. In the posterior scenario, the peak positive pressures at the proximal side (lower brainstem) are all more than 235 kPa. Therefore, subjects experiencing a posterior blast wave with the impinging BOP of more than 291 kPa are predicted to have serious cerebral contusion. The injury severities of the various cerebral locations including the cortex at the SSS, frontal cortex, occipital cortex, temporal cortexes, hypothalamus, and lower brainstem with respect to the five impinging BOPs at the blast incident side for the three exposure orientations are elucidated in Figure 7 (anterior exposure), Figure 8 (right lateral exposure), and Figure 9 (posterior exposure). Since the sharp transition from peak positive pressure to peak negative pressure may cause further damage to brain tissues than the uniform positive pressure or negative pressure, contusion predictions from the highest difference of the peak positive to negative pressure occurring within 1 ms are also made for the same locations by comparing the peak-to-peak pressure with the peak pressure based contusion criterion, and are illustrated in Figure 10 (anterior exposure), Figure 11 (right lateral exposure), and Figure 12 (posterior exposure).

The lateral blast exposure is predicted to be the most injurious orientation to the cortex among the three exposure orientations for the same blast severity, while the posterior exposure is the most injurious orientation to the lower brainstem. The lower brainstem is shown to be particularly vulnerable to contusion from bTBI because the pressure waves are ultimately funneled into the foramen magnum and the vertebral column for every blast scenario, and therefore subject the inferior cerebellum, medulla and spinal cord to large pressure fluctuations. Although the positive peak pressure responses along the brainstem are similar to that of the cortex that is proximal to the blasts in the right lateral and posterior orientations, they create additional risks for injuries because the superficial veins along the anterior surface of the brainstem are likely to be vulnerable to injury. The posterior fossa area with the large peak-peak pressure differences contains the intracranial portion of the vestibulo-cochlear and trigeminal nerves, which, if damaged, could contribute to the dizziness, hearing loss and headache that were observed acutely with mild blast TBI [14]–[15]. The intracranial pressure injury index of Ward et al. [40] was stated as applicable to cerebral contusion, so, for the injuries of the spinal cord, this criterion may not apply. Therefore, this pressure-based injury criterion is not used in the present study for injury severity analysis of the spinal cord. A detailed quantitative assessment of spinal cord injury severity will be discussed once a widely recognized injury criterion becomes available.

In the literature of impact TBI, relative motion between the skull and brain has been validated as a cause of subdural hemorrhage during the impact or acceleration events [8],[11]. However, in the present study, it is found that the relative displacement between the skull and brain is minimal (<0.01 mm, not shown) during the whole simulation time. Therefore, unlike the TBIs induced by impact or acceleration, primary bTBI is conjectured to not be caused by the relative motion between the skull and brain. The combination of this fact and the foregoing discussions of the pressure distribution pattern of bTBI consolidates our conclusions that the bTBI injury mechanism is attributed primarily to the propagation of the intracranial pressure wave induced by the direct transmission of the blast wave into the cranium and that the "coup-contrecoup" injury mechanism observed for impact- or acceleration-induced TBIs [13],[43] does not apply to primary bTBI.

Similar to the present study, Taylor and Ford [36] also predicted that the gray-white matter junction and the inferior regions such as brainstem had high von Mises stresses and that the von Mises stresses of the brain continued developing high levels through the whole simulation time. By investigating the maximum shear stress, Chafi et al. [4] it was predicted that high shear stress was initially in the cortical region at the coup site, and then propagated to other areas inside the brain, showing a different pattern than that of the present study. Chafi et al. [4] also found that the midbrain sustained a small amount of shear stress, which was in agreement with the present study. Chafi et al. [4] predicted that both the coup and contrecoup sites had the highest shear stress, and that the axonal injury could occur. However, highest shear stress is not predicted at the contrecoup site in the present study. Moore et al. [25] and Ganpule et al. [10] did not report the shear stress data.

Among all the three scenarios, the anterior scenario has the highest values of peak von-Mises stresses at the lower brainstem/medulla oblongata. The right lateral scenario has two primary intracranial sites (right temporal cortex and lower brainstem/inferior cerebellum within the posterior fossa) experiencing high-level von-Mises stresses with similar magnitudes. Similar to the anterior scenario, the posterior scenario has high-level von-Mises stress at the lower brainstem site, but the magnitudes are lower. Shear stress is proposed as the primary injury predictor of axonal injury for impact TBI in the study of Zhang et al. [43], who estimated the tolerable von-Mises stress levels within the brainstem to be 6.0 kPa, 7.8 kPa, 10.0 kPa for 25%, 50%, 80% probability of mild TBI. The highest value (2.81 kPa) of von-Mises stress in the present study is much lower than this blunt axonal injury criterion, therefore, it is hypothesized that von-Mises stress may not be the primary injury causation of blast TBI as opposed to intracranial pressure. Blast axonal injury is possible due to a stress or strain type other than von-Mises stress. However, without the direct evidence from a rigorous clinical study or a tissue-level quantitative study relating blast axonal damage to a specific stress or strain level, it is hard to completely determine the primary bTBI causation.

In summary, a full human head FE model and a numerical blast domain model is implemented to explore three scenarios of blast wave-head interaction simulations of effects of horizontal blast waves from three principal directions. Five blast wave-head simulations of 250 g, 300 g, 350 g, 400 g, and 450 g TNT charges at a one meter distance are conducted for each of the three scenarios. The sensitivities of the intracranial mechanical responses to blast intensities and exposure orientations are studied. The time-lapse pressure distribution of the brain reveals that the intracranial pressure wave propagates from the proximal blast side to the distal side for any blast orientation. At most of the cerebral locations, the pressure-time histories have an “impulse-like” pattern, which is very obvious at the proximal cerebral sites to the blasts. During the process of intracranial wave propagation from the proximal side to the distal side, attenuation of the pressure amplitude is observed, and the highest pressure levels are found to be at the parts of the brain that are proximal to blast for any blast orientation. This conclusion of brain pressure distribution of bTBI emphasizes that the attention of medical treatment and the design of protection devices should be addressed to consider the most probable cerebral region to sustain damage. The present study predicts that von Mises stress related axonal injury would not occur under any of the current blast scenarios. Since both high level pressures and shear stresses occur in the brainstem or the spinal cord during the blast events, future research should be focused on the detailed injury mechanisms of the brainstem and the spinal cord in bTBI, i.e. to perform the microscopic biomechanical analysis of the blast-induced injury there in order to evaluate the combinatorial effect of the pressure impulse and high sustained shear stress. There is also a research need of the blast axonal injury criterion for a more profound prediction of blast axonal injury. In the future, more geometric details can be added to the head model such as the falx and tentorium cerebelli, and the separation of white matter, gray matter, cerebellum, and brainstem. The separate constitutive modeling of the white matter and gray matter will be considered once the separate geometric modeling between them is accomplished.
